# Closed Incision Negative Pressure Wound Therapy in the Management of a Complex Fasciotomy Wound in a Pediatric Patient

**DOI:** 10.7759/cureus.7413

**Published:** 2020-03-25

**Authors:** Gordon Lee, Patrick C Murray, Ian G Hasegawa

**Affiliations:** 1 Orthopedics, John A. Burns School of Medicine, Honolulu, USA; 2 Orthopedic Surgery, University of Hawaii, Honolulu, USA; 3 Orthopedic Surgery, John A. Burns School of Medicine, University of Hawaii, Honolulu, USA; 4 Orthopedic Surgery, Queen's Medical Center, Honolulu, USA

**Keywords:** compartment, syndrome, pediatric, forearm, fracture, fasciotomies, negative pressure wound therapy, wound vac

## Abstract

Acute compartment syndrome (ACS) is a known entity that most often occurs in the setting of trauma in both adult and pediatric patients. Fasciotomy remains the gold standard treatment for relieving intracompartmental pressures but is associated with significant complications. Significant variability exists regarding fasciotomy wound management and closure. We present the only known case report on use of circumferentially applied negative pressure wound therapy instill and dwell (NPWTi-d) followed by circumferentially applied closed incision negative pressure wound therapy (ciNPWT) for the soft tissue management of delayed ACS in a pediatric patient.

## Introduction

Acute compartment syndrome (ACS) in the pediatric population is a rare orthopaedic emergency with potentially devastating consequences if not treated appropriately. Children pose unique challenges in identification and management of compartment syndrome. An unreliable physical exam and communication barrier are some of the challenges encountered with pediatric patients (especially with children <3), making the diagnosis of acute compartment syndrome in children notoriously difficult and often delayed. Studies report rates as high as 40-50% of pediatric ACS undergoing delayed treatment (>24 hours) [1,2]. Significant delays in treatment of ACS in pediatric patients are associated with permanent disability, flail limb, and/or need for amputations [3].

Gold standard treatment for pediatric compartment syndrome is fasciotomies, which are associated with extended hospital stay, infection, and secondary soft tissue procedures such as primary closure and grafting procedures [4]. Appropriate wound management is therefore needed to minimize potential complications and morbidity. Previous studies indicate negative pressure wound therapy (NPWT) has beneficial effects on fasciotomy wound healing by reducing time to closure in adults; however, there is a paucity of literature on the utility of NPWT in managing pediatric ACS [5]. We present a case report on the novel use of circumferentially applied negative pressure wound therapy with instillation and dwell (NPWTi-d) followed by circumferentially applied closed-incision negative pressure therapy (ciNPWT) for the management of a complex fasciotomy wound in a pediatric patient.

## Case presentation

History of present illness

Our patient is a three-year-old boy who presented to an outside hospital after jumping and falling from a couch thus sustaining a right forearm injury. He was diagnosed with a right both bone forearm fracture (BBFF), which includes the radius and ulna bones. The injury was successfully closed reduced and splinted without complication. Over the course of two days, the patient’s mother reported the patient's complaints of pain became progressive. Additionally, she noticed the patient was no longer moving his hand, and he was unresponsive to touch along his fingers. He was taken back to the hospital where compartment syndrome was suspected. The diagnosis was confirmed with intracompartmental pressure readings. The patient subsequently underwent volar and dorsal forearm fasciotomies where questionably viable muscle was reported. He was started on intravenous cefazolin, and subsequently transferred to the pediatric hospital for definitive management.

ER presentation

**Figure 1:**
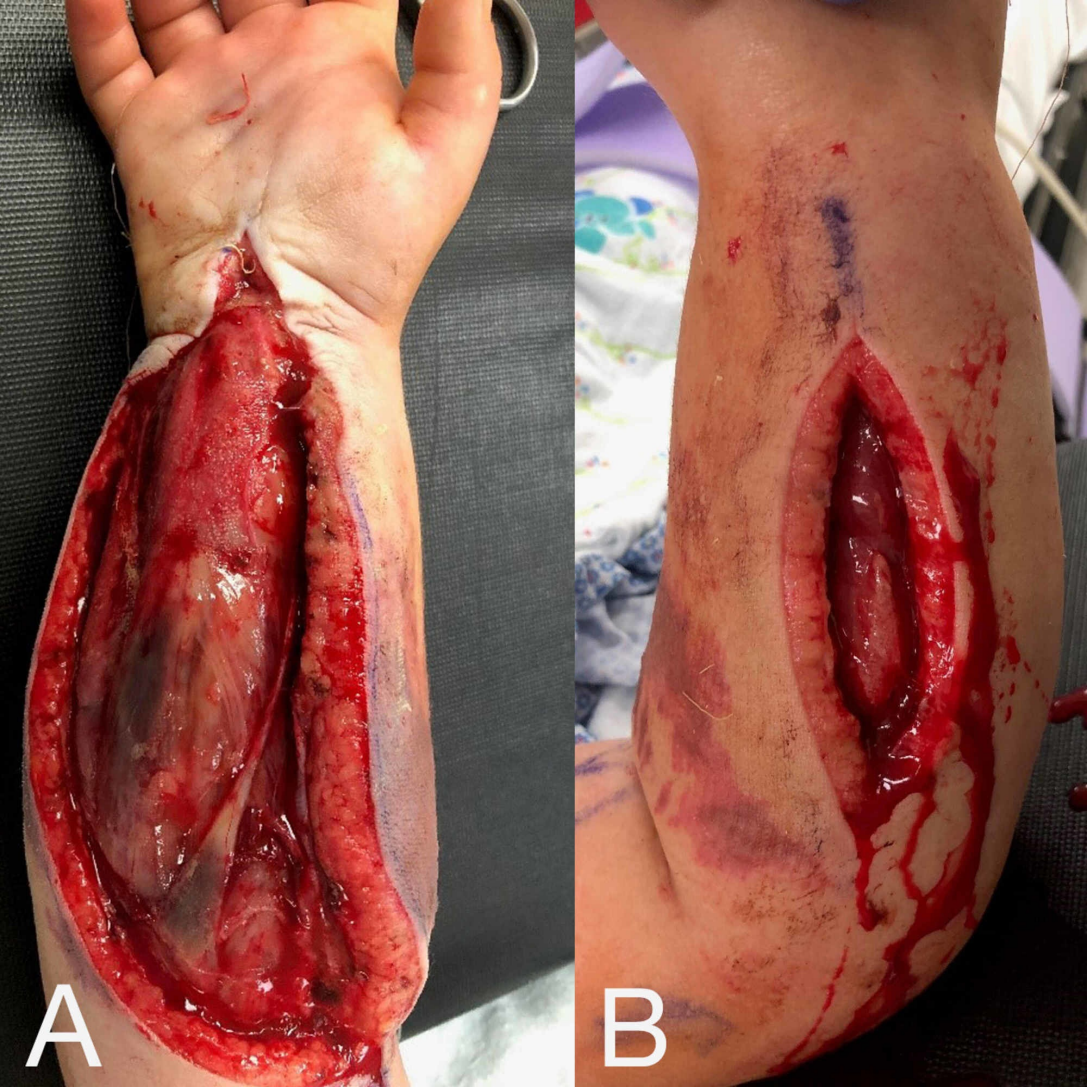
Initial presentation of right forearm fasciotomy wounds (A) Volar forearm fasciotomy site with myonecrosis noted proximally. At-risk and dusky skin is noted radially. (B) Dorsal forearm fasciotomy site.

Our patient was then seen in the emergency room at our facility where a repeat history and physical was performed. The comprehensive history and physical was unremarkable for new findings and was consistent with the previous findings prior to transfer in regards to the right upper extremity. Focused physical examination of the right upper extremity revealed persistent motor and sensory deficits of the median, radial, and ulnar nerves at the level of the wrist and fingers. The patient had no spontaneous movements of the right wrist or fingers and did not respond to touch. Volar and dorsal fasciotomy sites were clean without evidence of gross infection. Skin on the radial volar surface of the forearm presented with duskiness and surrounding erythema. Dusky muscle was noted at the proximal aspect of the volar and dorsal compartments (Figure 1A, 1B].

Laboratory work-up revealed creatine-kinase (CK) level of over 13,086 with no evidence of impaired renal function. Baseline electrolytes were within normal limits. Mild anemia was noted with hemoglobin level of 9, but without associated systemic or clinical symptoms, and therefore treatment was not indicated. The decision was made to admit the patient to the pediatric intensive care unit for hourly monitoring until surgery could be performed. Second look to ensure adequate release of forearm compartments, and repeat irrigation and debridement was indicated and would be performed as soon as possible. Intravenous fluids were started for kidney protection and CK clearance, and IV cefazolin was continued.

Hospital course

**Figure 2:**
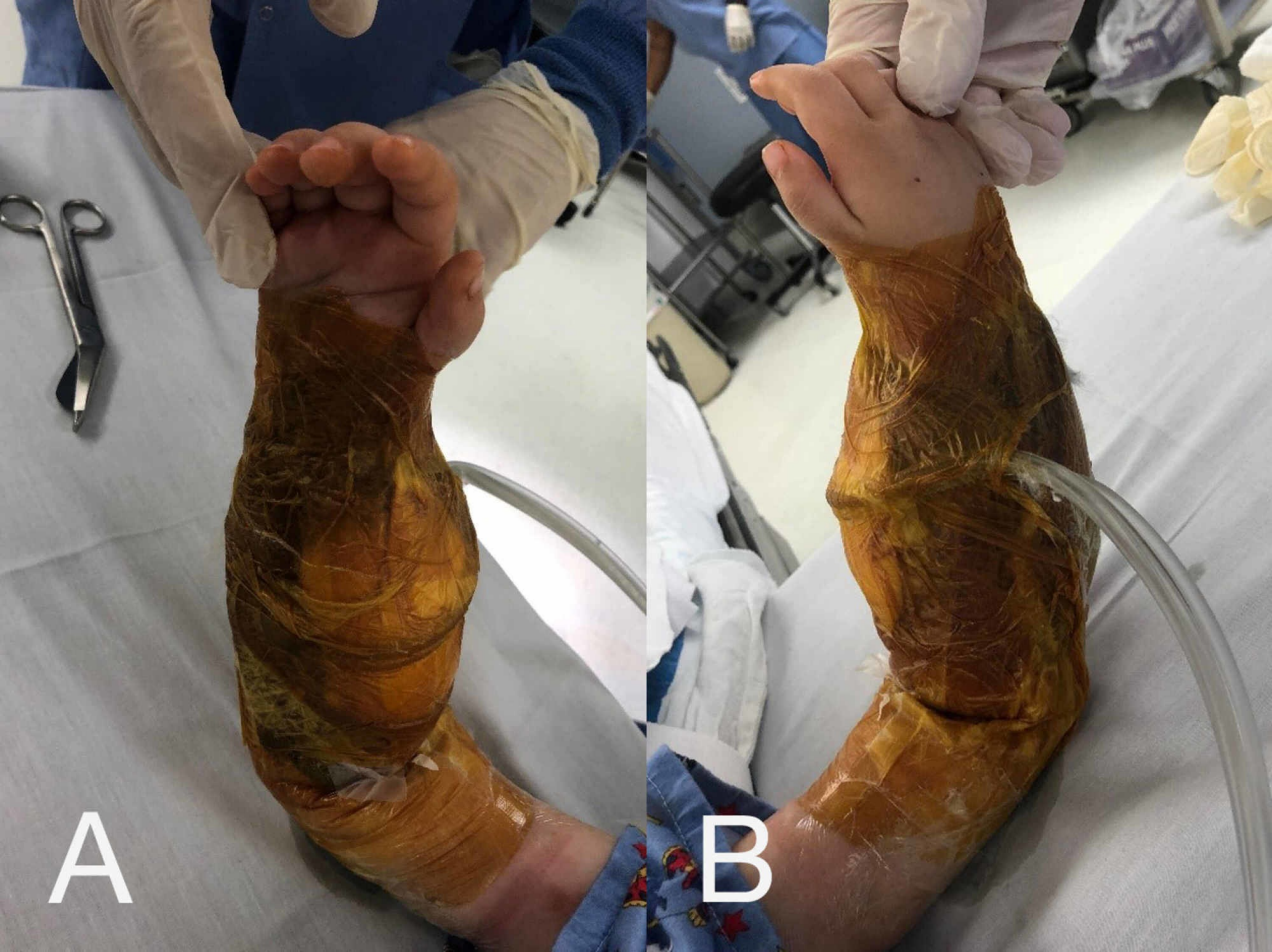
Circumferentially applied NPWTi-d dressing to right forearm fasciotomy wounds (A) Volar surface of right forearm with circumferentially applied NPWTi-d. (B) Dorsal surface with circumferentially applied NPWTi-d. Technique described below.

**Figure 3:**
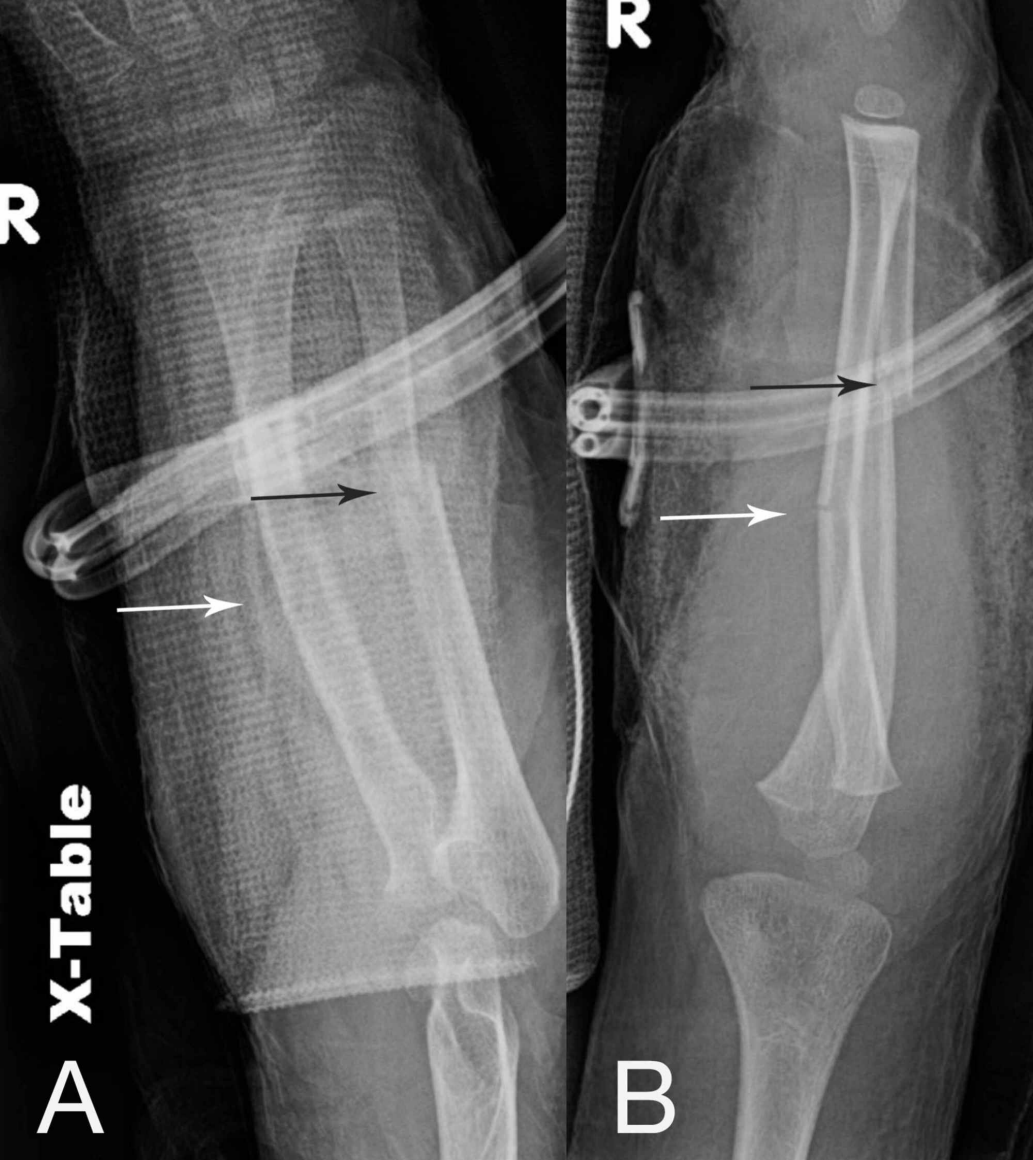
Plain radiographs demonstrating right both bone forearm fracture (A) AP and (B) lateral views of initial injury post initial irrigation/debridement and application of NPWTi-d. Black arrows demonstrate diaphyseal ulna fracture, and white arrows demonstrate diaphyseal radius fracture.

The patient was taken to the OR after admittance to the hospital for irrigation/debridement and reassessment of the right forearm. Dusky muscle was again noted at the proximal aspects of both dorsal and volar compartments, which was minimally responsive to electrocautery stimulation. We performed minimal debridement of the skin, muscle, and fascia. Clearly nonviable eschar was debrided, but any tissue with questionable viability was left alone. Application of a circumferential NPWTi-d using V.A.C. VERAFLO™ Instillation Therapy (Kinetic Concepts, Acelity, San Antonio, TX) was performed on the right forearm with wound vac settings set to -125 mmHg suction and instillation of Prontosan® Wound Irrigation Solution (B. Braun Medical Inc., Bethlehem, PA) (Figure 2A, 2B). Dwell time was set to 10 minutes cycled every 3 hours and the technique is described below. Cultures were taken and grew no organisms, and IV cefazolin was discontinued after this surgery. Repeat plain films were performed (Figure 3A, 3B), and the patient returned to the pediatric intensive care unit (PICU). While in the PICU, creatinine kinase was trended with continuous improvement and eventual resolution without evidence of any renal injury.

Circumferential NPWTi-d technique

First, an adequate and thorough irrigation and debridement is performed. Clearly necrotic/nonviable tissues are excised, and copious amounts of normal saline are used to irrigate the wounds using gravity. The wounds are then temporarily packed with dry lap sponges to keep the field and skin dry during adhesive application. ADAPTIC™ Non-Adhering Dressing (Acelity, San Antonio, TX) is placed on at-risk skin (in this case there was at-risk skin at the volar and radial aspect of the forearm). A single large sheet of clear wound vac adhesive from the V.A.C. VERAFLO CLEANSE CHOICE™ Dressing package is then used to wrap around the limb as a base layer to protect the perilesional and exposed skin from having direct sponge contact and minimize maceration. Extra care is taken to ensure adhesive is not creating tension on the soft tissues. Scalpel and/or scissors are then used to cut out the shape of the wounds from the layer of adhesive.

Lap sponges are removed. The honeycomb contact layer and thinner cover layer are then cut to the shape of wounds and placed in the wound bed without overstuffing. The thickest sponge cover layer is then cut axially to reduce its width by half. These are then laid in a circumferential pattern around the forearm over the base layer of adhesive applied to the skin (being sure there is no direct contact between sponge and skin). The sponge is then captured with any remaining clear adhesive. If needed, additional wound vac reinforcement is obtained utilizing 3M™ Ioban™ (3M Healthcare, St. Paul, MN). The included SENSAT.R.A.C.™ pad with instillation tubing is then applied over the area with the most theoretical bacterial bioburden (volar forearm site in this case). A cycle is run to establish correct seal, and the volume of the chosen instillation fluid is then chosen by utilizing the Fill Assist feature. We determine volume by filling until shadowing of the included gray sponge is clearly visualized. At our institution we generally opt for a 10-minute soak time cycled every 3 hours. Gauze is then placed under the tubing around the pad, and another layer of adhesive is placed on top to minimize the chance of the pad being pulled off during mobilization and/or transfers. A soft dressing and/or splint is then placed over the wound vac depending on injury and case characteristics.

Hospital course continued

On postoperative day (POD) 3, the patient was switched from Prontosan to a normal saline solution for instillation, and returned to the operating room on POD6. The wound vac was taken down, and fasciotomy sites were examined. Some duskiness to the muscle was still observed, however, there were signs of increased responsiveness to electrocautery. It was noted that the dusky skin on the radial volar forearm had developed into a fracture blister, which spontaneously unroofed when the wound vac was taken down.

Minimal excisional debridement was performed, repeat cultures were taken, and the patient underwent primary closure of both dorsal and volar wounds. A ciNPWT circumferential dressing was then placed without complication. The patient became febrile to 103.5 on POD0 with associated tachycardia and tachypnea. He was immediately started on empiric vancomycin and meropenem. On POD1, the intraoperative cultures taken from the fasciotomy and fracture sites became positive for enterococcus faecalis. The pediatric infectious disease team was consulted. Once sensitivities were finalized, the patient’s IV antibiotic regimen was tailored to ampicillin only.

**Figure 4:**
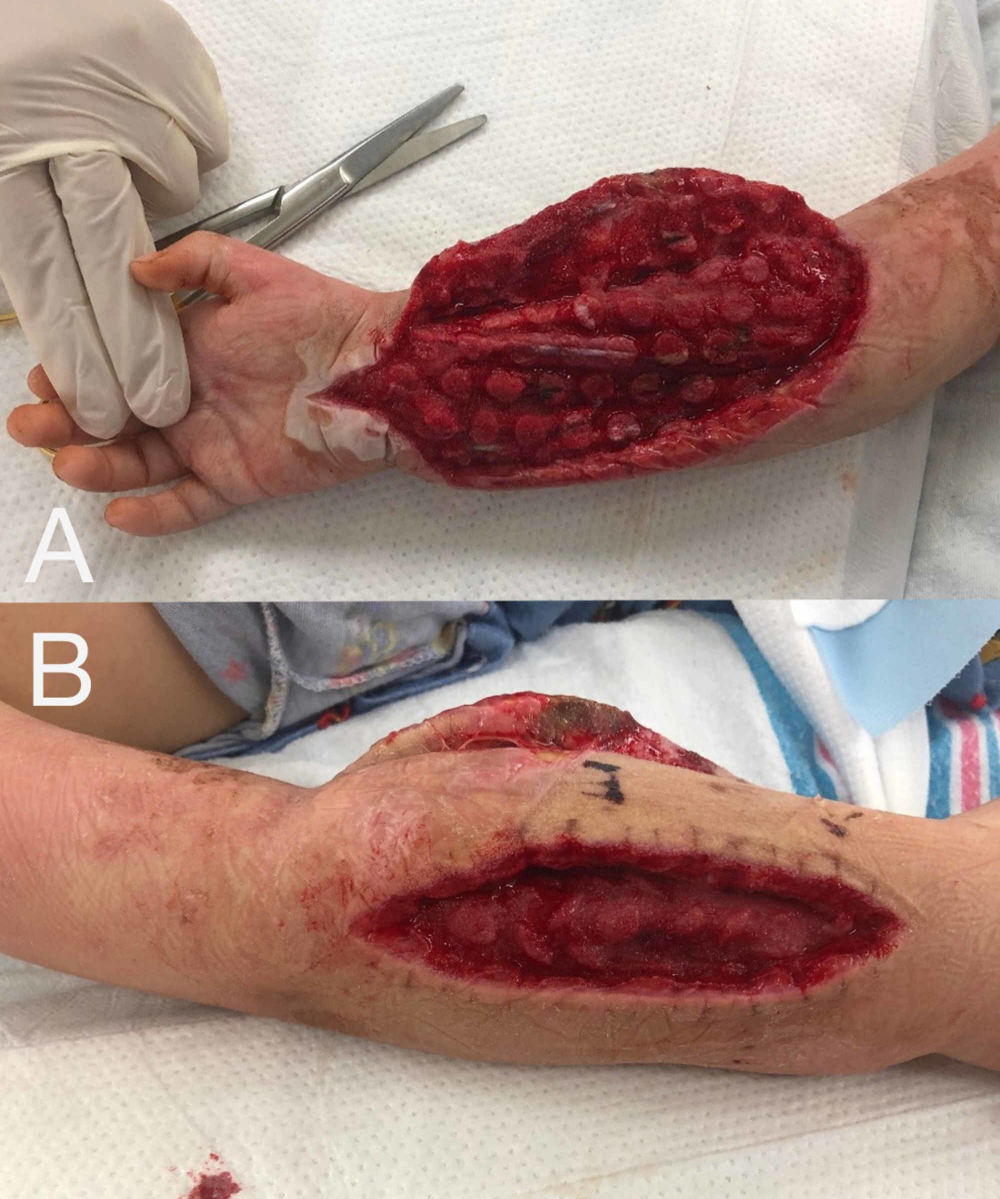
Improving appearance of volar and dorsal compartments (A) Volar forearm musculature with resolved myonecrosis and well perfused and viable musculature. (B) Improved dorsal forearm musculature appearance.

The patient required three additional operative procedures (repeat irrigation/debridements and circumferential NPWTi-d exchange) before negative cultures were confirmed and his incisions were ultimately closed. Each subsequent visit to the operating room showed progressive improvement in muscle viability with increased bleeding, improved color, and greater contractility (Figure 4A, 4B).

**Figure 5:**
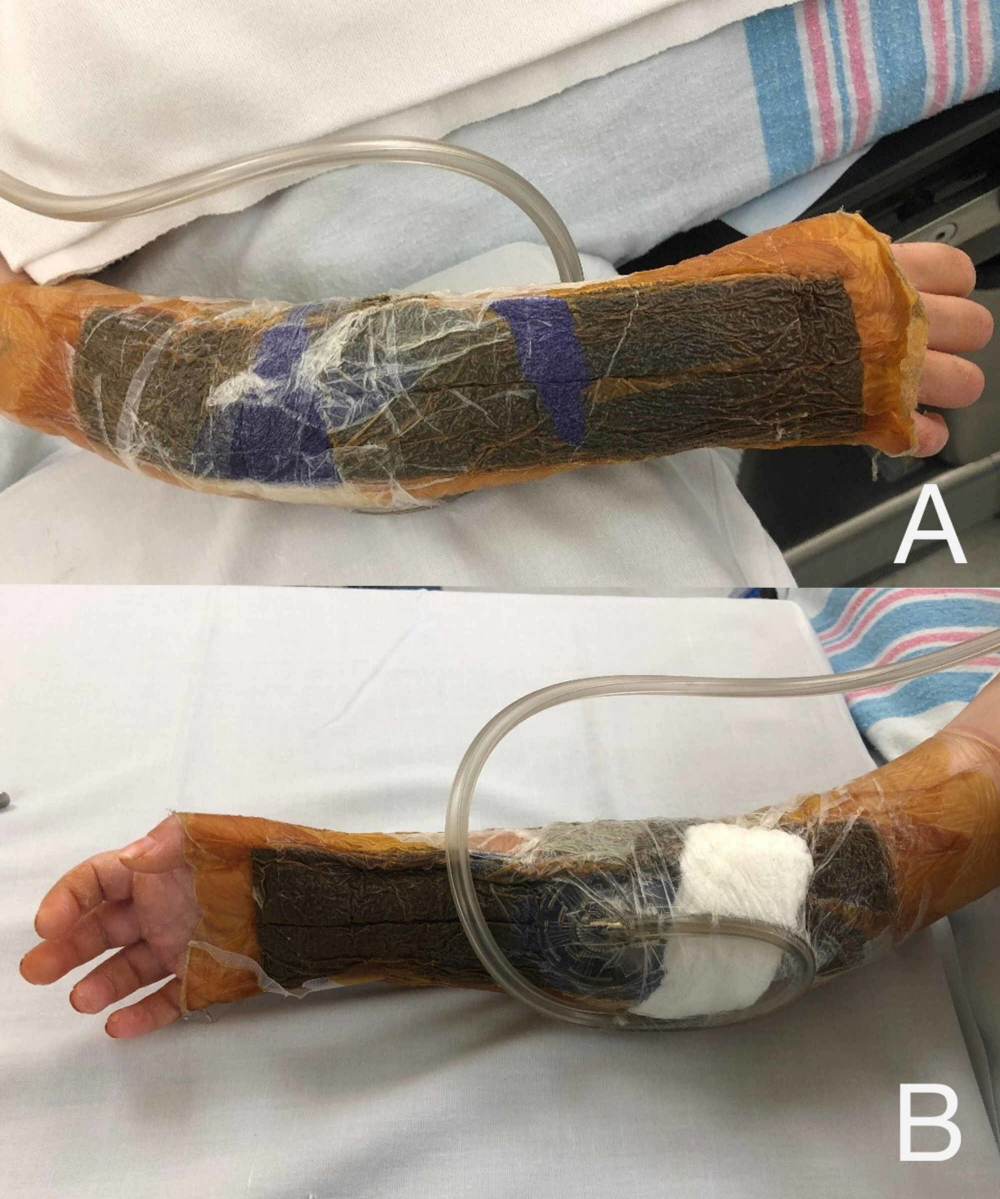
Circumferentially applied ciNPWT after primary closure of fasciotomy sites (A) Dorsal and (B) volar aspect of right forearm.

**Figure 6:**
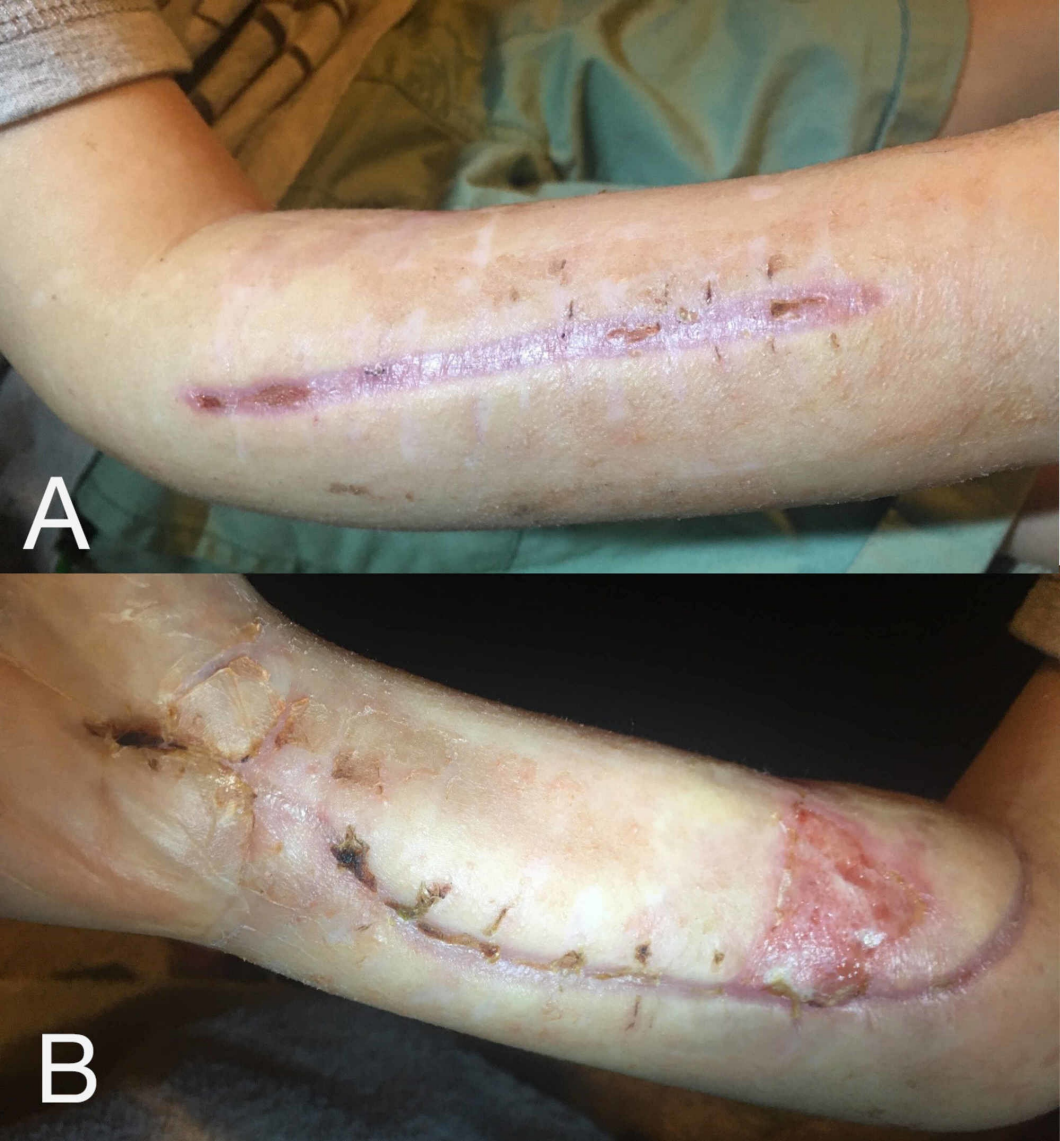
Healed fasciotomy sites (A) Dorsal and (B) volar fasciotomy sites completely healed with linear scar formation. Appropriate healing without dehiscence, infection, or need for grafting. Resolving volar skin ulcer from previously compromised skin at the proximal radial aspect is seen.

At the final irrigation/debridement and wound closure, vastly improved soft tissue envelopes and healthy muscle were noted. Primary closure was attempted and achieved with some difficulty, and a circumferential ciNPWT dressing utilizing PREVENA PLUS™ CUSTOMIZABLE™ System (Acelity, San Antonio, TX) was applied intraoperatively (Figure 5A, 5B). This circumferential vac was exchanged after one week and taken down altogether after an additional week. Fasciotomy sites were observed to be well approximated with excellent healing and no evidence of dehiscence or infection. Dorsal and volar wounds went on to heal without complication (Figure 6A, 6B).

Antibiotics (IV ampicillin only) were stopped prior to discharge. After discussion with the pediatric infectious disease team, no oral antibiotic therapy was pursued given the negative cultures and improvement in serial inflammatory markers. Local wound care for the resolving fracture blister was taught to the patient’s caregivers, and the patient was discharged to home with close follow-up arranged.

Follow-up

At the patient's most recent follow-up, linear scars from the fasciotomy sites persisted but were improved in appearance. The patient had complete bony union of his fractures and exhibited some return of movement at the level of the wrist and hand. He still exhibits sensory nerve deficits at the level of the hand. Progression of nerve healing is being monitored by a physiatrist and results have been promising. The most recent nerve conduction study demonstrates significant improvement of the right median and radial nerves. The prognosis of the right ulnar nerve remains guarded. Further operative procedures to address ulnar nerve deficit may be pursued in the future.

## Discussion

Acute pediatric compartment syndrome is a rare and potentially devastating condition. If further complicated by delayed diagnosis/treatment and evidence of irreversible myonecrosis/nerve damage, treatment can become challenging with no universally accepted guidelines. Recent clinical practice guidelines published by the American Academy of Orthopaedic Surgeons (AAOS) recommend against fasciotomies in cases of delayed diagnosis in adults [6]. There is evidence that myonecrosis present at fasciotomies is associated with increased wound complications such as infection, amputation, and need for repeat necrotic muscle debridement (Abstract: Hill G, Marsh JL, Buckwalter J. Necrotic Muscle at Fasciotomy. OTA Annual Meeting; October 17, 1997). However, these guidelines are not applicable to the pediatric population. Previous studies have shown that even in cases of delayed diagnosis and treatment, children still benefit from surgical decompression. Excellent outcomes have been reported as high as 87% [2]. In this case, because of our patient’s young age and perceived propensity to heal, delayed fasciotomies were performed.

Fasciotomies are the definitive treatment for compartment syndrome. Although necessary, they can be associated with significant morbidity and complications such as skin necrosis, dehiscence after closure, need for grafting procedures, infection, and extended hospital stays. Overall complication rates after fasciotomy have been reported as high as 30% with infection rates reaching up to 20% [7,8]. Our patient had multiple risk factors (myonecrosis at time of fasciotomy, delayed fasciotomies, previous antibiotic exposure) for infection and despite appropriate management, he developed a wound infection due to enterococcus faecalis.

Enterococcus faecalis is a known nosocomial infection that is becoming more of a worrisome entity as virulent strains become more prevalent. Risk factors for infection include exposure to previous antibiotics, repeat surgical procedures, ICU stay, and immunocompromise [9,10]. Treatment usually consists of an IV antibiotic regimen based on culture sensitivities. In our patient, a three-week course of intravenous ampicillin was enough to eradicate infection and improve abnormal labs. In addition to receiving IV antibiotics, our patient underwent multiple washouts and NPWT dressing exchanges.

Advent of NPWT has altered the way fasciotomy wounds are managed. Studies support that NPWT causes microdeformation in wounds, promoting angiogenesis and improving blood flow to the wound [11]. We posited that this effect would greatly improve our chances to salvage as much muscle/nerve/and skin as possible. Furthermore, NPWT has been shown to help with soft tissue edema by reducing vessel permeability and thus fluid build-up in the tissues [12]. Because of our belief blood flow would improve to the damaged muscle, we opted to do minimal sharp excisional debridement of skin only and apply a circumferential NPWTi-d. The instill and dwell NPWT dressing was chosen due to its ability to reduce bioburden of bacteria and aid in the clearance of infection [13]. With the support of NPWTi-d, the infection at the fasciotomy sites was cleared and wound edema was managed, allowing for successful primary closure.

Primary wound closure after fasciotomy is ideal for multiple reasons. It allows for reestablishment of normal muscular pressures and tension, theoretically allowing for peak muscle forces. It also spares the patient from having a cosmetically unpleasant and frequently insensate scar. Additionally, grafting procedures performed on pediatric patients are less than ideal, often requiring extended hospital stays for monitoring, dressing changes, pain control, and bolster removal [14,15].

Despite the advantages associated with primary closure, it is frequently impossible or unsafe to perform. Grafting procedures are often required after fasciotomy with rates of 25%-88% reported in literature [1,4]. Factors associated with significantly diminished likelihood for primary closure and need for grafting include delayed fasciotomies and repeat procedures [16,17]. Weaver et al. observed primary closure was unachievable in all fasciotomies requiring >2 irrigation/debridement procedures. In our case, we were able to obtain primary closure with the assistance of NPWT despite delayed fasciotomies and multiple visits to the operative theatre.

After primary closure, a ciNPWT was applied that offered multiple advantages. Laboratory studies have shown negative pressure dressings decrease perilesional wound edema and increase the force needed to cause wound dehiscence of closed incisions [18,19]. Furthermore, the ciNPWT dressing is applied intraoperatively under sterile conditions. Therefore, it acts as a physical barrier to outside sources of contamination. These factors (edema management, angiogenesis, mechanical tension off-loading, and barrier protection) combine to create optimal conditions for wound healing. Additionally, another advantage to using ciNPWT is it eliminates the need for multiple painful dressing changes on the floor. This enhances patient comfort and reduces the psychological and somatic burden on patient, staff, and family.

## Conclusions

Ultimately, the entirety of the patient’s forearm musculature and most neurovascular structures were salvaged. As of his most recent follow-up, the patient’s radius/ulna fractures have completely united and linear scar appearance continues to remodel. Furthermore, the patient also has shown clinical and electrophysiologic evidence of early reinnervation to the forearm. We expect him to continuously improve with time.

Overall, we believe this novel application of a circumferential ciNPWT in our patient played an integral part in his care. The advantages of NPWTi-d and ciNPWT used in this case likely maximized wound healing, increased comfort, and minimized complications. Additionally, with the support of the ciNPWT, the patient was able to avoid a prolonged hospital stay, increased healthcare costs, and morbidity related to a skin-grafting procedure. In conclusion, our case report demonstrates ciNPWT can be advantageously utilized in the management of complex and at-risk pediatric wounds.
